# Screening Tools Used by Clinical Pharmacists to Identify Elderly Patients at Risk of Drug-Related Problems on Hospital Admission: A Systematic Review

**DOI:** 10.3390/pharmacy8020064

**Published:** 2020-04-10

**Authors:** Amanda Brady, Chris E. Curtis, Zahraa Jalal

**Affiliations:** 1 Pharmacy Department, Sligo University Hospital, Sligo F91 H684, Ireland; amanda_mandy_jenkins@yahoo.co.uk; 2 School of Pharmacy, Institute of Clinical Sciences, College of Medical and Dental Sciences, University of Birmingham, Birmingham B15 2TT, UK; z.jalal@bham.ac.uk

**Keywords:** clinical pharmacy, risk assessment tools, frail elderly, hospital care

## Abstract

In recent years, a number of studies have examined tools to identify elderly patients who are at increased risk of drug-related problems (DRPs). There has been interest in developing tools to prioritise patients for clinical pharmacist (CP) review. This systematic review (SR) aimed to identify published primary research in this area and critically evaluate the quality of prediction tools to identify elderly patients at increased risk of DRPs and/or likely to need CP intervention. The PubMed, EMBASE, OVID HMIC, Cochrane Library, PsychInfo, CINAHL PLUS, Web of Science and ProQuest databases were searched. Keeping up to date with research and citations, the reference lists of included articles were also searched to identify relevant studies. The studies involved the development, utilisation and/or validation of a prediction tool. The protocol for this SR, CRD42019115673, was registered on PROSPERO. Data were extracted and systematically assessed for quality by considering the four key stages involved in accurate risk prediction models—development, validation, impact and implementation—and following the Checklist for the critical Appraisal and data extraction for systematic Reviews of prediction Modelling Studies (CHARMS). Nineteen studies met the inclusion criteria. Variations in study design, participant characteristics and outcomes made meta-analysis unsuitable. The tools varied in complexity. Most studies reported the sensitivity, specificity and/or discriminatory ability of the tool. Only four studies included external validation of the tool(s), namely of the BADRI model and the GerontoNet ADR Risk Score. The BADRI score demonstrated acceptable goodness of fit and good discrimination performance, whilst the GerontoNet ADR Risk Score showed poor reliability in external validation. None of the models met the four key stages required to create a quality risk prediction model. Further research is needed to either refine the tools developed to date or develop new ones that have good performance and have been externally validated before considering the potential impact and implementation of such tools.

## 1. Introduction

A drug-related problem (DRP) has been defined as ‘an event or circumstance involving drug therapy that actually or potentially interferes with the desired health outcome’ [1]. DRPs can cause serious harm, hospital admissions, increased health care costs and even death and are known to be prevalent in elderly patients both in the community and in the hospital setting [2]. DRPs include medication errors, adverse drug reactions (ADRs), and adverse drug events (ADEs) [3]. Elderly (or older) people, defined as those aged 65 years and over [4,5] with multiple co-morbidities receiving multiple medicines for management/treatment of long-term illness are prone to adverse drug reactions and are likely to be more susceptible to adverse drug events in general [2]. The “oldest-old” are a subgroup of elderly people, defined by the WHO as those aged 80 years and older [5]. The reported percentages of ADRs for all hospitalized patients vary between 2.4% and 10.9% [6], with a higher incidence of ADRs in older people, as they are prescribed significantly more drugs than younger individuals, and older people are nearly seven-times more likely to be hospitalized due to an ADR compared to younger people [6,7]. A number of studies have aimed to determine the risk factors associated with ADEs, ADRs and/or DRPs which may help to identify patients at risk of such events and prioritise these (high risk) patients for intervention [7,8]. The authors of a previous systematic review (SR) concluded that failure to consider risk prediction in a clinical setting can result in poor care and many guidelines have recommended incorporating life expectancy into clinical decisions to help target services to those who might benefit the most [9], such as in decisions to discontinue breast, colon and prostate cancer screening based on age and life expectancy [10,11,12].

A simplistic definition of inappropriate prescribing is that it is “The practice of administering medications in a manner that poses more risk than benefit, particularly where safer alternatives exist” [13]. Inappropriate prescribing encompasses potentially inappropriate medications (PIMs) (misprescribing and overprescribing) and potential prescribing omissions (PPOs) or underprescribing [14,15]. Inappropriate prescribing is highly associated with an increased risk of adverse drug events especially in older patients with poly pharmacy who are more vulnerable [15]. In their review of inappropriate prescribing, O’Connor et al. commented that “given the complexity of prescribing decisions in the elderly a more holistic definition of inappropriate prescribing should encompass the assessment of older persons’ prescription medications in the context of their multiple co-morbidities, complex medication regimes, functional and cognitive status, treatment goals and life expectancy” [14]. Whilst not exclusive to the elderly, the prescribing of potentially inappropriate medications to older people has been shown to be highly prevalent, with rates ranging from 12% for community-dwelling elderly to 40% in nursing home residents [16]. Inappropriate prescribing is associated with adverse drug events [16]. A number of tools have been developed to measure and assess medication appropriateness in older people which can be measured and assessed by using, for example, the Medication Appropriateness Index (MAI). In addition, inappropriate prescribing criteria sets such as the STOPP/START criteria can be applied to medication lists. However, these tools can be time consuming to utilise, which currently limits their use in routine clinical practice [17].

It is well recognised both within Europe and internationally that there is a need to prioritise pharmaceutical care [18,19]. Hospital clinical pharmacists are a limited and expensive resource, making routine screening of all Emergency Department patients by pharmacists unsustainable [20]. Within the National Health Service (NHS) in the United Kingdom, it is recognised that “Clinical pharmacists cannot review all patients every day” [19] and that “to allocate clinical pharmacist resource where it is most needed, patients’ pharmaceutical care needs must be assessed and prioritised accordingly” [19]. In America, a $0.5 million research grant was awarded by the American Society of Health-System Pharmacists to develop and validate a pharmaceutical complexity scoring tool [18]. Pharmacists cannot identify and manage all drug-related problems but need to prioritise and “identify those drug-related problems for which management or prevention would result in the greatest benefit for as many patients as possible” [21]. In a recently published article looking at prioritising pharmaceutical care, the authors commented that there is an “urgent need for pharmacy departments to prioritise which patients need direct pharmaceutical care on a daily basis” [18].

There is limited published literature about the current imperfect use of pharmaceutical care priority screening tools in UK hospitals [18]. In recent years, a number of researchers have looked at identifying risk factors for drug-related problems (DRPs) and developing screening and acuity tools to help prioritise clinical pharmacist workflow, and research continues to develop such tools both in the UK and abroad [18,22]. A SR published in 2016 found 38 studies (that met their inclusion criteria) that identified measurable risk factors associated with adult hospital inpatients that potentially lead to a pharmaceutical intervention [23]. Stevenson et al. conducted a SR of risk prediction models used to predict ADRs in older adults [7]. They included studies that developed and validated an ADR prediction model for use in patients over 65 years old, using a multivariable approach in the design and analysis; only four studies met the inclusion criteria for the SR, emphasising the deficit in published research on this topic in this subgroup of patients (hospital inpatients aged over 65 years) [7]. Stevenson et al.’s SR included only four studies, and additional studies have been published since 2014. Furthermore, a quality assessment of studies could not be generated in Stevenson et al. [7]. This paucity of evidence was the driver to carry out a systematic review of the literature to (1) identify any existing primary research that involves the development and/or utilisation and/or validation of a prediction tool or risk score system to identify those patients most likely to experience DRPs and/or benefit from hospital pharmacist intervention, and (2) critically evaluate the quality of the identified research. In particular, this SR aimed to identify and focus on any research that includes or is specific to elderly patients (aged over 65 years old).

## 2. Materials and Methods 

### 2.1. Data Resources

A systematic search of research articles published in peer-reviewed health care-related journals was performed. The following databases were searched from inception until April–May 2018 with no date restrictions applied: PubMed, Embase, OVID HMIC, Cochrane Library, Psychinfo, CINAHL PLUS, Web of Science, and ProQuest. 

These databases were selected based on previous SRs conducted by pharmacist researchers with similar aims. The titles of all papers (n=1030) were screened and, if the title suggested that the paper might be relevant to the topic (prediction tools and/or risk factors for DRPs), the abstracts (where available from search databases) were reviewed by the principal researcher (AB). Following this review of titles and abstracts, full-text publications were accessed for all potentially relevant papers and evaluated through intensive reading by the principal researcher. Details of all sourced full-text articles were tabulated to allow validation of a final list of citations and for a final list of included papers to be drawn up. Details of any articles for which there were queries were also tabulated and were referred to two independent reviewers (CC and ZJ). The reviewers considered the queries separately and then the three researchers discussed each individual query paper and reached consensus on whether it should be included in the SR. From the search of the eight databases, eight papers were identified that met the inclusion criteria.

A number of additional papers (11 further papers) were identified for inclusion in this SR (these papers were found in the initial scoping search and in the databases, and duplicates were removed). An internet search using the search engine Google Scholar was used as part of a scoping exercise when the principal researcher was initially considering conducting research in this area in order to gauge the amount of published research on this topic. From this search, a relevant systematic review published in 2014 was identified assessing the use of risk prediction models to predict adverse drug reactions in older people [7]. The systematic review (which was not itself included in this SR, as it did not meet the inclusion criteria) identified four relevant papers; these four papers were sourced and reviewed to determine whether they were relevant for inclusion in this systematic review. 

An additional paper was also identified whilst trying to locate the full-text article of one of the papers included in the aforementioned SR, namely a paper by Tangiisuran B. [24], which led to the identification of another paper by Tangiisuran B. et al. [25] reporting the same research, namely the BADRI risk prediction model; these two papers describe the same research and have been considered as a single piece of research in this SR.

Another four papers were identified having been referenced or mentioned in one of the 8 papers from the search of the medical databases. Three further papers were identified through keeping up to date with research publications and from a review of relevant conference proceedings.

**Figure 1:**
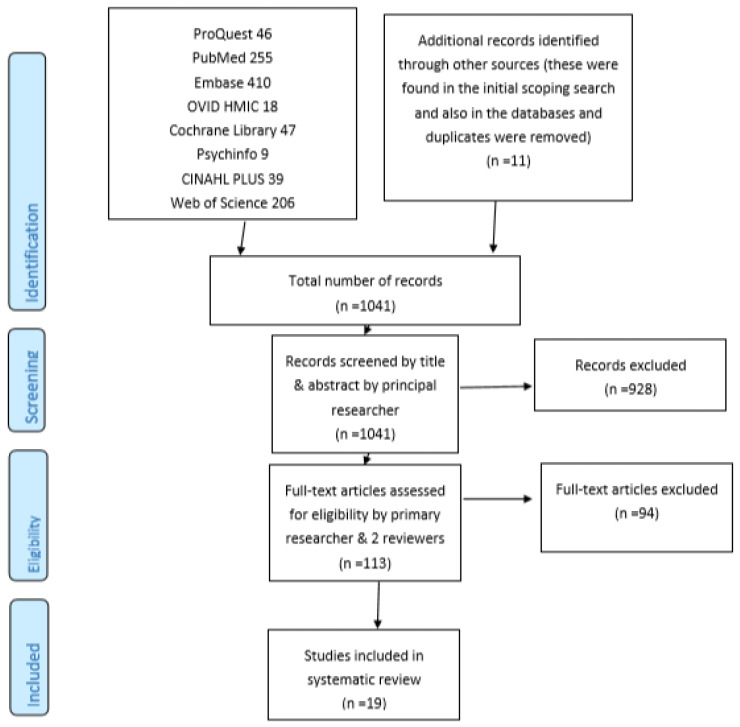
PRISMA flow diagram.

A total of 19 papers were identified and included in the SR through the systematic search of eight medical databases and other methods described as summarised in Figure 1.

### 2.2. Data Extraction

Results were extracted and tabulated according to the papers included in the SR, population characteristics, sample size, study setting, whether the study examined at development, utilisation and/or validation of prediction tool, study outcomes, risk tools and performance of risk tools.

### 2.3. Search Terms and Search Strategy

PICOS is a framework designed to make the process of defining and designing a research question easier, where PICOS stands for: P Population/Patient, I Intervention, C Comparison, O Outcome, and S Study Design [26]. The author referred to the principles of the PICOS method to formulate a search strategy and select keywords relevant to the current research. 

In view of the structural and content-related differences between the databases, particularly between the indexing and thesauri/controlled vocabulary used by PubMed (Medical Subject Headings, MeSH) and EMBASE (Emtree) a number of different search terms and combinations of terms were used. A combination of MeSH/Emtree and free-text search terms were used. The search terms included synonyms and various combinations of the following keywords “elderly”, “pharmacist”, “hospital”, “risk”, “risk factor” and “risk assessment,” with the combinations used reflecting the specific search capabilities of the different databases. For example, for those databases identifying a comparatively large number of articles from a search using the terms “elderly”, “pharmacist” and “hospital”, additional terms relating to risk were added to narrow the search to focus upon more relevant articles. Keywords not listed as MeSH or Map Terms were searched as phrases using the free-text search mode. A further list of search terms was generated by referring to a previous review with similar aims [7]. The reference list of relevant papers was also searched in order to identify any additional studies. Duplicate articles were removed if they were found in the different databases. 

The search strategy appears in File S1, PubMed search strategy. This was supported by use of a checklist File S2 PRISMA 2009 checklist to ensure that PRISMA principles were followed during the process.

The following combination of search terms was used when conducting the scoping exercise (already described) on Google Scholar: +risk tool +elderly +drug-related problems. 

Figure 1 summarises the number of potentially relevant papers that were identified and the final number included in the SR, based on (a simplified version of) the PRISMA flow diagram.

### 2.4. Inclusion and Exclusion Criteria

#### 2.4.1. Paper Inclusion Criteria

Inclusion criteria for the SR included studies published in the English language, primary research/studies including (but not exclusively) patients aged 65 years and over, inpatients in secondary or tertiary care centres (hospitals), medically admitted patients, where interventions take place in the hospital setting, all primary research and research for which the full-text article was available for review.

#### 2.4.2. Paper Exclusion Criteria

Exclusion criteria for the SR included systematic reviews; literature reviews; meta-analyses; summary articles; discussion articles; conference proceedings; editorials; surgically admitted patients; studies solely in patients in specialist care settings, e.g., intensive care or psychiatry (but not geriatric/older persons wards); studies looking at specific areas, e.g., blood glucose or HbA1c control or dementia; studies conducted in community pharmacy, primary care settings, outpatients or attending outpatient clinics (e.g., diabetes, heart failure clinics), those in ambulatory care and patients (solely) aged <65 years; studies where one or more of the interventions took place in the home/community setting; studies that focussed solely on interventions at the point of or following hospital discharge/transition. The SR excluded proposals for research (e.g., published research protocols) for which results had not been published (in journals included in the selected databases)/were not available through the databases used and research where only the abstract was available for review. In addition, studies using a set of arbitrary or predetermined factors incorporated in a tool/scoring system that were not developed or tested with any statistical analysis methods (such as regression analysis) to determine relevance of the variables were excluded.

#### 2.4.3. Critical Appraisal and Data Extraction for Included Studies

The studies identified via the searches were initially reviewed by the principal researcher considering the four key stages involved in accurate risk prediction models—development, validation, impact and implementation—as described by Petrovic M. [27]. To assess the bias and quality of the included studies in a systematic manner, the Checklist for the critical Appraisal and data extraction for systematic Reviews of prediction Modelling Studies (CHARMS) was used by the principal researcher to critically appraise the studies. The items extracted using the checklist were then tabulated into Excel spreadsheets—the format of which was influenced by the tabulated results included in a recently published systematic review relevant to this topic by Falconer N. et al. [28]. 

The protocol for this SR, CRD42019115673, was registered on PROSPERO [29].

## 3. Results

Nineteen papers were included in the SR, and the citations are included in Table 1.

### 3.1. Included Papers

Full details of the data extracted from the included studies are included in the Supporting Information, Table S1.

### 3.2. Population Characteristics: Age

All of the studies were conducted in the hospital setting and, as per inclusion criteria, in adult patients. Seven of the studies were carried out exclusively in elderly patients (aged 65 years and over) [25,30,31,32,41,43,44] and only the very elderly (aged 80 years and over) were represented in a further study [33]. Two studies [18,38] did not report the age demographics of the study participants (although participants were adults). The remaining studies were conducted in adult patients (including but not exclusively elderly patients) [3,20,34,35,36,37,39,40,42]. The majority of the studies presented either the mean or median age for participants, with the mean age of participants varying from 51.4 years [20] to 86.7 years [33], showing significant variations between the participants of the various studies.

### 3.3. Population Characteristics: Gender

There were significant differences in the gender profiles for participants in the differing studies, with four studies [18,30,38,42] not reporting this characteristic; in the remaining fifteen studies, female participants represented 46%–72% of the sample populations, so there were significant differences between the study populations with regard to this, with differences also observed between those included in the development and individual validation studies carried out by the same researchers, e.g., females accounted for 49% of participants in the first validation study conducted by Kaufmann et al. in 2018, compared to 61% of participants in the second validation study after the questionnaire was revised [37].

### 3.4. Population Characteristics: Ethnicity

Ethnicity was only reported in two of the studies [25,38], with 88% white-British ethnicity reported in one study [38] and 51.1% European, 23% Pacific Island decent, Maori and 13.6% ‘others’ (25) in the second study.

The other study was conducted in New Zealand and the population that the hospital served was described as being “an ethnically diverse population largely consisting of people of Maori and Pacific descent, with a high proportion of the population living in the most socio-economically deprived communities” [38]. This highlights significant differences between the populations where the two studies that reported participant ethnicity were conducted (Brighton, UK compared to New Zealand).

### 3.5. Population Characteristics: Number of Study Participants

The number of participants included in each study varied significantly from 35 patients [18] to 10,807 [35]. 

### 3.6. Development/Modelling, Utilisation or Validation of Assessment Tools

Different studies examined the development/modelling, utilisation or validation of an assessment tool and combinations of these phases. Validation of the majority of studies was internal, whilst four provided external validation [20,30,32,43], and one of these studies included both internal and external validation [25]. The majority of studies used separate populations to develop and subsequently validate their tools, whilst two studies [31,33] used the same populations and used bootstrapping techniques to test the developed tool and a third used internal cross validation methods [40]. Overall, nine of the studies involved the development (or adaptation of a draft version of a tool) and internal validation of the assessment tool [3,18,31,34,40,41,42,44]. One study involved the development and external validation of a tool [30], whilst another involved the development, internal and external validation of a tool [25]. One study [20] only described the development of an assessment tool (which was subsequently utilised in a further study; see below [35]); another study described the development and subsequent utilisation (rather than validation) of a tool [38], four studies described the utilisation or internal validation of a previously developed tool [35,36,37,39], and two studies provided external validation (only) of the previously developed GerontoNet ADR Risk Score [32,43].

### 3.7. Study Setting (Country/Countries)

The majority of the studies (74%) were conducted in European centres [3,18,25,30,31,32,33,34,36,37,41,42,43,44], and the remaining studies were conducted in New Zealand [38,39], Canada [20,35] and the US [40]. In two of the European-based studies [25,30], the development phase took place in Europe and the developed tools were then externally validated within centres in four European countries (and also internally validated in one of the studies [25]). The GerontoNet ADR Risk Score was developed and externally validated in Europe [30] and subsequently externally validated in two further European studies [32,43].

### 3.8. Primary Outcomes of the Studies

The primary outcomes for the studies varied significantly. Five studies assessed the incidence (or rate) of ADRs [25,30,32,43,44] and another three studies looked at the incidence (or rate) of ADEs [20,31,41]. There were also subtle differences between studies with the same broad primary outcomes with respect to definitions/interpretations—for example, one study looked at the incidence of “non-trivial ADRs” [44] and another specified that their primary outcomes were ADEs that required a change in medical therapy, diagnostic testing, consultation, or hospital admission [20]. The primary outcomes for the remaining studies were more specific/individual to each study and included the incidence of medication errors per patient [42], prescribing errors (PEs) [36], medication discrepancies on medication reconciliation at admission and Pes [39], time to rehospitalisation or death during the year following discharge [33], the number of DRPs per admission [3], the rate of potentially avoidable hospital readmission at 30 days [40] and the rate of pharmacist interventions [34]. These variations in the primary outcomes (and even differences in the exact definitions included within these primary outcomes) made it difficult to compare or collate the results and findings of the different studies, as they were not measuring the same parameters. 

### 3.9. Number of Risk Factors Included in Risk Scores/Tools

The number of risk factors identified by and included in the risk scores/tools ranged from three [36,40,42] to thirty eight [38]. One study described the development of two clinical decision rules (CDRs) containing seven and four risk factors respectively [20]; in a subsequent study by the same researchers, a modified ADE CDR was used which was composed of five risk factors [35]. One study identified thirty eight flags for inclusion as part of an Assessment Risk Tool (ART) [38]; in the subsequent validation study reported in 2017, twenty five of these ART flags were included—four of which were found to be significantly associated with at least one unintentional medication discrepancy [39].

### 3.10. Identification of Risk Factors

The risk factors included in the various studies and tools/scores were identified via a number of methods—these included clinical judgement, from literature review (alone), patient characteristics/variables and by combinations of methods. For example, one study identified possible risk factors from available study data (demographic and clinical details) and previously established risk factors from published reports [44]. In another study, five consecutive steps (literature search, Delphi process, construction of algorithm, calibration of algorithm, and prospective pilot study) were used to develop the algorithm [42].

### 3.11. Statistical Analysis

Statistical analysis was conducted and reported to determine the performance of the various tools/models in seventeen of the nineteen studies [3,18,20,25,30,31,32,33,34,36,37,39,40,41,42,43,44], but there was variation in the extent and types of statistical analyses conducted. In the other two studies [35,38], the development of a tool was described but validation of the tool was not conducted (38); statistical analysis was conducted to determine the value of medication review by pharmacists of patients identified by the CDR but not to evaluate the performance of the CDR itself [35].

### 3.12. Methodologies for Development of Point Scores

Different methods were used in the various studies to develop the point scores for the tools/models described. In 15 of the studies, the methodology described the weighting system used for the different variables included in the scores—of which, 14 were individualised for the different variables [3,30,31,32,33,34,36,38,39,40,41,42,43,44], whilst one assigned arbitrary weighting to the variables [25]. Four of the 14 studies with individualised weighting were based on theoretical weighting [36,42] or determined via group consensus [38,39]; the exact methodology used for determining weighting was unclear in one study [41] (although odds ratios and beta coefficients were calculated and included in the write up). In the remaining nine (of the 14) studies, the methodologies described the calculation and use of odds ratios, beta coefficients and regression coefficients to determine weightings of individual variables. Four studies did not report on the calculation of weightings of variables in risk scores; in one study, no score was calculated [37], whilst the levels of risk were assigned by meeting set criteria for following an algorithm in the remaining three studies [18,20,35].

### 3.13. Performance of Risk Score/Tool/Model: Sensitivity, Specificity and/or Discriminatory Ability

The majority of the studies reported on the sensitivity, specificity and/or discriminatory ability of the risk score/tool/model. Sensitivity and specificity were reported in six of the studies [20,25,30,37,41,42], and discriminatory ability (including AUC/AUROC/AUCROC, C-statistics) or overall accuracy was reported in thirteen of the studies [3,25,30,31,32,33,34,39,40,41,42,43,44] including four studies, where sensitivity, specificity and discriminatory ability were all reported [25,30,41,42]. There were four studies which did not report sensitivity, specificity or discriminatory ability/overall accuracy of the scores/tools [18,35,36,38]. These results are summarised in Table 2.

### 3.14. Sample Size and Power of Study

The events per variable (EPV) ratio is often used to assess the adequacy of the sample size when developing a risk model. The EPV ratio “is calculated by using the number of outcomes divided by the number of candidate predictor variables in the development cohort” and a minimum EPV ratio of 10 is usually recommended {28]. The results show that only two studies had adequate sample sizes using the EPV ratio>10 rule [3,30]. The remaining studies that involved the development of a risk model/tool either did not contain an adequate sample size [25,33] or the details were unclear or not reported in these studies. In the case of one study [38], the tool consisting of 38 variables or risk factors was developed from consensus opinion rather than modelling methods and so the EPV ratio was regarded as not applicable in this instance.

In the validation studies, only four studies [3,32,35,39] were sufficiently powered (with >100 events), and the remaining validation studies were either underpowered or there was not sufficient information to accurately assess this parameter [28].

### 3.15. Quality of Included Studies

Six (32%) of the included studies described how missing data were handled [20,25,35,37,40,43]. In the remaining studies, it was either unclear or not reported how missing data were handled. From the papers describing the studies, study outcomes were assessed blinded in six studies [18,20,35,36,39,42]. It was either unclear or not reported whether study outcomes were assessed blinded in the remaining studies. Only two of the studies [3,30] that included the development of a tool using modelling methods (excluding, for example, the development of a tool by consensus methods rather than modelling [18]) reported a sufficient sample size using the “rule of thumb” of the EPV ratio>10 that is commonly adhered to by researchers when developing prediction tools [28]. In the validation studies or those studies with a separate validation arm, only four [3,32,35,39] reported a sufficient event rate to suggest that the (validation) study was adequately powered (with >100 events reported). In the remaining studies, the sample size or powering was either insufficient or not clearly reported. 

## 4. Discussion

The population characteristics, methodologies, primary outcomes and presentation of results of the nineteen studies included in this SR vary significantly, making quantitative synthesis of their findings and meta-analysis unsuitable for this review. Instead, narrative synthesis of the findings was conducted and presented.

The results of this SR do not currently support the use of any of the models identified in routine clinical practice, similar to the findings of a previous SR [7]. Only four of the nineteen studies included any external validation [25,30,32,43]. External validation would provide confidence that a particular model’s predictive ability is reliable across different populations and settings. Even in the four studies which incorporated external validation, there were limitations to usefulness of the models/scores involved as outlined below 

The sensitivity of a predictive model measures the ability of the model to correctly predict (identify) individuals who will experience the outcome (e.g., who have an ADR) (true positive), whilst specificity is a measure of the true negative rate of the model, measuring the ability of the model to correctly predict those individuals who will not experience the outcome (e.g., an ADR). Whilst a satisfactory level of sensitivity of 80% was reported for the BADRI model, its low specificity of 46% meant that “the model may incorrectly label patients ‘at risk of an ADR’ who will not ordinarily go on to experience such an event” [25]. 

The GerontoNet ADR Risk Score (developed by Onder et al.) showed comparable discriminatory ability to the BADRI model (with AUROC of 0.71 in the development phase for the GerontoNet ADR Risk Score and 0.74 for internal validation of the BADRI model) but the sensitivity for the GerontoNet ADR Risk Score was lower than that for the BADRI model (68% and 80% respectively); the specificity of both models was low but the specificity was higher for the GerontoNet ADR Risk Score (at 65%) compared to the BADRI model (at 55%) [25,30].

External validation of the GerontoNet ADR Risk Score [32] identified an AUC value of 0.62 (on admission) and also reported that the score incorrectly classified 38% of patients as being at low risk of ADRs [32], showing poor performance of the score [43].

A second published external validation for the GerontoNet ADR Risk Score [43] reported that AUC values of 0.64 and 0.69 were calculated to predict (1) ADRs probably or definitely related to drug use and (2) type A ADRs (type A (intrinsic) reactions are those that are usually predictable from the known pharmacology of a drug), respectively. From the researchers’ own definitions of model performance, these AUC values show poor performance of the GerontoNet ADR Risk Score. In particular in subpopulations of older hospitalised patients, the GerontoNet ADR Risk Score was found to have fair to good diagnostic accuracy, with AUC values of 0.70–0.79 and 0.80–0.89. It was concluded that the GerontoNet ADR Risk Score could be adopted for use in older patients belonging to the specific subpopulations; these findings suggest that the GerontoNet ADR Risk Score may not be suitable for routine clinical use in the general elderly (65 years and over) hospital in-patient population for which it was originally developed [43].

### Limitations

There are a number of possible limitations to this SR. The search strategy used to identify relevant studies included “pharmacist” as an essential search term. Whilst this was deemed appropriate, as the focus of the SR was prioritising patients for pharmacist review, it is possible that other research that identified risk prediction models for elderly patients experiencing DRPs could have been excluded if they did not include a pharmacist in the study methodology; such studies may have provided useful insight into factors and models to predict the likelihood of DRPs in elderly in-patients. To minimise the potential for “missing” relevant studies, the PICOS framework was used to formulate the search strategy, and all three researchers extensively discussed the choice of search terms to be used. Due to the heterogenous nature of the methodologies and patient demographics, the outcomes and reporting of results, it was not possible to conduct a meta-analysis and instead a qualitative analysis was conducted. However, the CHARMS checklist was used to try to systematically assess the quality of the included studies and minimise subjectivity. It is recognised that it was difficult to assess bias due to the relatively small number of studies (insufficient), heterogeneity and the lack of preregistered protocols [45].

## 5. Conclusions

The findings from this systematic review show that a number of tools have been developed for use in the (acute) hospital setting for assessment of the risk of older people experiencing drug-related problems, including ADRs and AEs. The tools developed to date vary in their complexity, outcome measures and how their performance has been validated. There is no definitive validated assessment tool which is in widespread use for this group of patients. None of the tools identified in this SR “satisfied the four key stages in the creation of a quality risk prediction model” (7)—development, validation, impact and implementation. Whilst research to date has focused on the development and validation of such tools, further work is needed to assess the potential impact of utilising the tools and their implementation. 

## Figures and Tables

**Table 1:** Table showing the papers included in this systematic review (SR).

Author	Tool
Tangiisuran B. et al. [25] (note: this research was also reported in PhD thesis [24] but only counted as one piece of research in this SR)	BADRI model
Onder G. et al. [30]	GerontoNet ADR Risk Score
Trivalle C. et al. [31]	A geriatric score
O’Connor M.N. et al. [32]	GerontoNet ADR Risk Score
Alassaad A. et al. [33]	The 80+ score
Suggett E. [34]	Risk scores to identify patients who were at high risk of (hospital) pharmacist intervention (no specific name for score stated by authors)
Hohl C.M. et al. (2017) [35]	Clinical decision rules
Hohl C.M. et al. (2012) [20]	Clinical decision rules
Bonnerup D.K. et al. [36]	MERIS score
Kaufmann C.P. et al. (2018) [37]	Drug-Associated Risk Tool (DART)
Falconer N. et al. (2014) [38]	Assessment of Risk Tool (ART)
Falconer N. et al. (2017) [39]	Assessment of Risk Tool (ART),
McAuliffe L. et al. [40]	MEDCOINS score
McElnay J.C. et al. [41]	A predictive model for adverse drug events (ADEs) in elderly patients (no specific name for model stated by authors)
Urbina O. et al. [3]	A score that quantifies the risk of a DRP during hospital admission (no specific name for score stated by authors)
Hickson R.P. et al. [18]	Pharmaceutical assessment screening tool (PAST)
Saedder E.A. et al. [42]	MERIS score
Petrovic M. et al. [43]	GerontoNet ADR Risk Score
O’Mahony D. et al. [44]	The adverse drug reaction risk in older persons (ADRROP) prediction scale

**Table 2:** Table showing the reported sensitivity, specificity, discriminatory ability and overall accuracy of the risk scores/tools used in each study.

Author	Sensitivity	Specificity	Other			
			Discriminatory Ability: AUROC/AUCROC	Discriminatory Ability: C-statistic	Overall Accuracy	Other
Tangiisuran B. et al.	80.0% for internal validation; 84% for external validation	55.0% for internal validation; 43% for external validation	0.74 (95%CI = 0.68–0.79) for internal validation; 0.73 (95%CI = 0.66–0.80) for external validation			
Onder et al.	68% (development)	65% (development)	0.71 (95% CI = 0.68–0.73) (development) 0.70 (95% CI = 0.63–0.78) (validation)			
Trivalle et al.	n/r	n/r	0.70 (95% CI = 0.635–0.74)			
O’Connor et al.	n/r	n/r	0.62 (95% CI = 0.57–0.68) on admission (lower on subsequent days, days 5 & 10 post-admission)			
Alassaad A. (&et al)	n/r	n/r		0.71		
Suggett E.	n/r	n/r	0.607 for main data; 0.616 for validation data			
Hohl et al (2017)	n/r	n/r	n/r	n/r	n/r	
Hohl et al (2012)	1) More sensitive rule (ADE rule) 96.7% (95%CI = 91.8–98.6%)	1) 40.3%				
	2) More specific rule (Adverse Drug Reaction Rule) 90.8% (95%CI = 81.4–95.7%)	2) 59.1%				
Bonnerup et al.	n/r	n/r	n/r	n/r	n/r	
Kaufmann et al.	67% average (range = 21–100)	88% average (range = 27–100)	n/r			
Falconer et al. (2014)	n/r	n/r	n/r	n/r	n/r	
Falconer et al. (2017)	n/r	n/r	0.72 (95%CI = 0.66–0.78) to predict at least one unintended medication discrepancy; from score from 2 flags (>8 regular admission medicines and readmission within 30 days of discharge)			
McAuliffe et al.	n/r	n/r		0.65 (95%CI = 0.60–0.70)		Hosmer-Lemeshow goodness-of-fit = 0.99
McElnay et al.	41% (validation)	69% (validation)			63%	
Urbina et al.	n/r	n/r	0.778 (95% CI = 0.768–0.789) (training set); 0.776 (95%CI = 0.759–0.792) (validation set)			Hosmer-Lemeshow goodness-of-fit = non-significant (p = 0.13) (validation set)
Hickson et al.	n/r	n/r	n/r	n/r	n/r	n/r
Saedder et al.	0.64 (for final version of MERIS)	0.75 (for final version of MERIS)	0.76, 0.87, 0.74, 0.66 for final MERIS score in different populations within study			
Petrovic et al.	n/r	n/r	0.64 (95%CI = 0.55–0.74) to predict ADRs probably or definitely related to drug use; 0.69 (95%CI = 0.60–0.77) for predicting Type A ADRs; subgroup analysis: AUC = 0.70–0.79 and 0.80–0.89			
O’Mahony et al.	n/r	n/r	0.623 (95% CI = 0.598–0.665) (derivation cohort); 0.592 (95%CI = 0.532–0.652) (vallidation cohort)			

n/r = not relevant.

## Supplementary Materials

The following are available online at https://www.mdpi.com/2226-4787/8/2/64/s1, File S1 PUBMED search strategy, File S2 PRISMA Checklist, and Table S1: Summary of results.

## References

[1] Pharmaceutical Care Network Europe The PCNE Classification V 9.0. https://www.pcne.org/upload/files/334_PCNE_classification_V9-0.pdf.

[2] NHS East & South East England Specialist Pharmacy Services. Medicines Related Problems on Admission to Hospital—The Evidence 2014. https://www.sps.nhs.uk/wp-content/uploads/2014/04/Medicines_related_problems_on_admission_the_evidence_Apr14_Vs2_JW.pdf.

[3] Urbina O., Ferrández O., Grau S., Luque S., Mojal S. (2014). Marin-Casino Mea. Design of a score to identify hospitalized patients at risk of drug-related problems. Pharmacoepidemiol. Drug Saf..

[4] World Health Organization Proposed Working Definition of an Older Person in Africa for the MDS Project 2002. https://www.who.int/healthinfo/survey/ageingdefnolder/en/.

[5] World Health Organization Men Ageing and Health—Achieving Health Across the Life Span 2001. https://apps.who.int/iris/bitstream/handle/10665/66941/WHO_NMH_NPH_01.2.pdf?sequence=1.

[6] Petrovic M., Somers A., Onder G., Stegemann S. (2016). Geriatric Pharmacotherapy: Optimisation Through Integrated Approach in the Hospital Setting. Developing Drug Products in an Aging Society.

[7] Stevenson J., Williams J., Burnham T., Prevost A., Schiff R., Erskine D., Davies J.G. (2014). Predicting adverse drug reactions in older adults; A systematic review of the risk prediction models. Clin. Interv. Aging.

[8] Alhawassi T., Krass I., Bajorek B., Pont L. (2014). A systematic review of the prevalence and risk factors for adverse drug reactions in the elderly in the acute care setting. Clin. Interv. Aging..

[9] Yourman L., Lee S., Schonberg M., Widera E., Smith A. (2012). Prognostic Indices for Older Adults A Systematic Review. JAMA J. Am. Med Assoc..

[10] Bibbins-Domingo K., Grossman D.C., Curry S.J., Davidson K.W., Epling J.W., García F.A.R., Gillman M.W., Harper D.M., Kemper A.R., Krist A.H. (2016). Screening for Colorectal Cancer: US Preventive Services Task Force Recommendation Statement. JAMA.

[11] Rodin M.B. (2000). Breast cancer screening in older women: American Geriatrics Society Clinical Practice Committee. J. Am. Geriatr. Soc..

[12] Wolf A., Wender R., Etzioni R., Thompson I., D’Amico A., Volk R.J., DeSantis C. (2010). American Cancer Society Guideline for the Early Detection of Prostate Cancer: Update 2010. CA A Cancer J. Clin..

[13] Reference.MD Inappropriate Prescribing. http://www.reference.md/files/D057/mD057970.html.

[14] O’Connor M.N., Gallagher P., O’Mahony D. (2012). Inappropriate prescribing: Criteria, detection and prevention. Drugs Aging.

[15] O’Mahony D., O’Sullivan D., Byrne S., O’Connor M.N., Ryan C., Gallagher P. (2014). STOPP/START criteria for potentially inappropriate prescribing in older people: Version 2. Age Ageing.

[16] Gallagher P., Barry P., O’Mahony D. (2007). Inappropriate prescribing in the elderly. J. Clin. Pharm. Ther..

[17] Lavan A.H., Gallagher P.F., O’Mahony D. (2016). Methods to reduce prescribing errors in elderly patients with multimorbidity. Clin. Interv. Aging.

[18] Hickson R.P., Steinke D.T., Skitterall C., Williams S.D. (2017). Evaluation of a pharmaceutical assessment screening tool to measure patient acuity and prioritise pharmaceutical care in a UK hospital. Eur. J. Hosp. Pharm. Sci. Pract..

[19] National Health Service (2014). Greater Glasgow and Clyde. Pharmacy Prioritisation and Referral. PostScript Acute.

[20] Hohl C.M., Yu E., Hunte G.S., Brubacher J.R., Hosseini F., Argent C.P., Singer J. (2012). Clinical decision rules to improve the detection of adverse drug events in emergency department patients. Acad. Emerg. Med. Off. J. Soc. for Acad. Emerg. Med..

[21] Bruchet N., Loewen P., Lemos J. (2011). Improving the Quality of Clinical Pharmacy Services: A Process to Identify and Capture High-Value? Quality Actions?. Can. J. Hosp. Pharm..

[22] El Hajji F.W., Scullin C., Scott M.G., McElnay J.C. (2015). Enhanced clinical pharmacy service targeting tools: Risk-predictive algorithms. J. Eval. Clin. Pract..

[23] Suggett E., Marriott J. (2016). Risk Factors Associated with the Requirement for Pharmaceutical Intervention in the Hospital Setting: A Systematic Review of the Literature. Drugs Real World Outcomes.

[24] Tangiisuran B. (2009). Predicting Adverse Drug Reactions in the Hospitalised Elderly. Ph.D. Thesis.

[25] Tangiisuran B., Scutt G., Stevenson J., Wright J., Onder O., Petrovic M. (2014). Development and Validation of a Risk Model for Predicting Adverse Drug Reactions in Older People during Hospital Stay: Brighton Adverse Drug Reactions Risk (BADRI) Model. PLoS ONE.

[26] Liberati A., Altman D.G., Tetzlaff J., Mulrow C., Gøtzsche P.C., Ioannidis J.P.A., Moher D. (2009). The PRISMA statement for reporting systematic reviews and meta-analyses of studies that evaluate healthcare interventions: Explanation and elaboration. Ann. Intern. Med..

[27] Petrovic M. Adverse drug reactions in older people and their prevention: The need for a new approach. Proceedings of the International Congress of The European Union Geriatric Medicine Society (EuGMS).

[28] Falconer N., Barras M., Cottrell N. (2018). Systematic review of predictive risk models for adverse drug events in hospitalized patients. Br. J. Clin. Pharmacol..

[29] Brady A., Curtis C., Jalal Z. (2019). Prediction Tools, Models and Risk Score Systems Used by Clinical Pharmacists in the Acute Hospital Setting to Identify Elderly Patients Most Likely to Experience Drug Related Problems and/or Benefit From Hospital Pharmacist Intervention: A Systematic Review of the Literature. CRD42019115673. PROSPERO. https://www.crd.york.ac.uk/prospero/display_record.php?ID=CRD42019115673.

[30] Onder G., Petrovic M., Tangiisuran B., Meinardi M.C., Markito-Notenboom W.P. (2010). Development and Validation of a Score to Assess Risk of Adverse Drug Reactions Among In-Hospital Patients 65 Years or Older. The GerontoNet ADR Risk Score. Arch. Intern. Med..

[31] Trivalle C., Burlaud A., Ducimetière P. (2011). Risk factors for adverse drug events in hospitalized elderly patients: A geriatric score. Eur. Geriatr. Med..

[32] O’Connor M.N., Gallagher P., Byrne S., O’Mahony D. (2012). Adverse drug reactions in older patients during hospitalisation: Are they predictable?. Age Ageing.

[33] Alassaad A., Melhus H., Hammarlund-Udenaes M., Bertilsson M., Gillespie U., Sundstrom J. (2015). A tool for prediction of risk of rehospitalisation and mortality in the hospitalised elderly: Secondary analysis of clinical trial data. BMJ Open.

[34] Suggett E.L. (2017). Risk Assessment of Patients in an Acute Trust as a Means of Directing a Clinical Pharmacy Service. Ph.D. Thesis.

[35] Hohl C.M., Partovi N., Ghement I., Wickham M.E., McGrail K., Reddekopp L.N., Sobolev B. (2017). Impact of early in-hospital medication review by clinical pharmacists on health services utilization. PLoS ONE.

[36] Bonnerup D.K., Lisby M., Saedder E.A., Sorensen C.A., Brock B., Andersen L., Nielsen L.P. (2016). Risk of prescribing errors in acutely admitted patients: A pilot study. Int. J. Clin. Pharm..

[37] Kaufmann C.P., Stampfli D., Mory N., Hersberger K.E., Lampert M.L. (2018). Drug-Associated Risk Tool: Development and validation of a self-assessment questionnaire to screen for hospitalised patients at risk for drug-related problems. BMJ Open.

[38] Falconer N., Nand S., Liow D., Jackson A., Seddon M. (2014). Development of an electronic patient prioritization tool for clinical pharmacist interventions. Am. J. Health Syst. Pharm. AJHP Off. J. Am. Soc. Health Syst. Pharm..

[39] Falconer N., Liow D., Zeng I., Parsotam N., Seddon M., Nand S. (2017). Validation of the assessment of risk tool: Patient prioritisation technology for clinical pharmacist interventions. Eur. J. Hosp. Pharm. Sci. Pract..

[40] McAuliffe L.H., Zullo A.R., Dapaah-Afriyie R., Berard-Collins C. (2018). Development and validation of a transitions-of-care pharmacist tool to predict potentially avoidable 30-day readmissions. Am. J. Health Syst. Pharm. AJHP Off. J. Am. Soc. Health Syst. Pharm..

[41] McElnay J.C., McCallion C.R., Al-Deagi F., Scott M.G. (1997). Development of a Risk Model for Adverse Drug Events in the Elderly. Clin. Drug Investig..

[42] Saedder E.A., Lisby M., Nielsen L.P., Rungby J., Andersen L.V., Bonnerup D.K., Brock B. (2016). Detection of Patients at High Risk of Medication Errors: Development and Validation of an Algorithm. Basic Clin. Pharmacol. Toxicol..

[43] Petrovic M., Tangiisuran B., Rajkumar C., van der Cammen T., Onder G. (2017). Predicting the Risk of Adverse Drug Reactions in Older Inpatients: External Validation of the GerontoNet ADR Risk Score Using the CRIME Cohort. Drugs Aging.

[44] O’Mahony D., O’Connor M., Eustace J., Byrne S., Petrovic M., Gallagher P. (2018). The adverse drug reaction risk in older persons (ADRROP) prediction scale: Derivation and prospective validation of an ADR risk assessment tool in older multi-morbid patients. Eur. Geriatr. Med..

[45] Ayorinde A.A., Williams I., Mannion R., Song F., Skrybant M., Lilford R.J., Chen Y.F. (2018). Assessment of publication bias in systematic reviews of health services and delivery research (Poster). J. Epidemiol. Community Health.

