# Supplementary material for: Screening Tools Used by Clinical Pharmacists to Identify Elderly Patients at Risk of Drug-Related Problems on Hospital Admission: A Systematic Review
Source: Pharmacy (Basel). 2020 Apr 10;8(2):64. doi: 10.3390/pharmacy8020064 (PMC7355869; doi:10.3390/pharmacy8020064)
Supplement: Supplementary file 1 [file pharmacy-08-00064-s001.zip › Supplementary files/File S1.docx]

**File S1: Pubmed search strategy**

("aged"[MeSH Terms] OR "aged"[All Fields] OR "elderly"[All Fields]) AND ("pharmacists"[MeSH Terms] OR "pharmacists"[All Fields] OR "pharmacist"[All Fields]) AND ("hospitals"[MeSH Terms] OR "hospitals"[All Fields] OR "hospital"[All Fields]) AND ("risk assessment"[MeSH Terms] OR ("risk"[All Fields] AND "assessment"[All Fields]) OR "risk assessment"[All Fields]) AND tools[All Fields]

("aged"[MeSH Terms] OR "aged"[All Fields] OR "elderly"[All Fields]) AND ("pharmacy"[MeSH Terms] OR "pharmacy"[All Fields] OR "pharmacies"[MeSH Terms] OR "pharmacies"[All Fields]) AND ("hospitals"[MeSH Terms] OR "hospitals"[All Fields] OR "hospital"[All Fields]) AND ("risk assessment"[MeSH Terms] OR ("risk"[All Fields] AND "assessment"[All Fields]) OR "risk assessment"[All Fields])

("aged"[MeSH Terms] OR "aged"[All Fields] OR "elderly"[All Fields]) AND ("pharmacy"[MeSH Terms] OR "pharmacy"[All Fields] OR "pharmacies"[MeSH Terms] OR "pharmacies"[All Fields]) AND ("secondary care"[MeSH Terms] OR ("secondary"[All Fields] AND "care"[All Fields]) OR "secondary care"[All Fields]) AND tools[All Fields]

("risk"[MeSH Terms] OR "risk"[All Fields]) AND tool[All Fields] AND ("aged"[MeSH Terms] OR "aged"[All Fields] OR "elderly"[All Fields]) AND drug[All Fields] AND related[All Fields] AND problems[All Fields]

("risk"[MeSH Terms] OR "risk"[All Fields]) AND tool[All Fields] AND ("pharmacists"[MeSH Terms] OR "pharmacists"[All Fields] OR "pharmacist"[All Fields]) AND ("aged"[MeSH Terms] OR "aged"[All Fields] OR "elderly"[All Fields]) AND drug[All Fields] AND related[All Fields] AND problems[All Fields]

("pharmacists"[MeSH Terms] OR "pharmacists"[All Fields] OR ("clinical"[All Fields] AND "pharmacist"[All Fields]) OR "clinical pharmacist"[All Fields]) AND drug[All Fields] AND related[All Fields] AND problems[All Fields] AND ("aged"[MeSH Terms] OR "aged"[All Fields] OR "elderly"[All Fields]) AND ("risk assessment"[MeSH Terms] OR ("risk"[All Fields] AND "assessment"[All Fields]) OR "risk assessment"[All Fields]) AND tools[All Fields]
